# Cost-Effectiveness of Outreach Strategies for Stool-Based Colorectal Cancer Screening in a Medicaid Population

**DOI:** 10.1089/pop.2021.0185

**Published:** 2022-06-07

**Authors:** Jordan J. Karlitz, A. Mark Fendrick, Jay Bhatt, Gloria D. Coronado, Sushanth Jeyakumar, Nathaniel J. Smith, Marcus Plescia, Durado Brooks, Paul Limburg, David Lieberman

**Affiliations:** ^1^ Division of Gastroenterology, Denver Health Medical Center and University of Colorado School of Medicine, Denver, Colorado, USA.; ^2^ Division of General Medicine and Center for Value-Based Insurance Design, University of Michigan, Ann Arbor, Michigan, USA.; ^3^ Chicago School of Public Health, University of Illinois, Chicago, Illinois, USA.; ^4^ Kaiser Permanente Center for Health Research, Portland, Oregon, USA.; ^5^ Maple Health Group, LLC, New York, New York, USA.; ^6^ Associate of State and Territorial Health Officials, Atlanta, Georgia, USA.; ^7^ Exact Sciences, Madison, Wisconsin, USA.; ^8^ Division of Gastroenterology and Hepatology, Mayo Clinic, Rochester, Minnesota, USA.; ^9^ Division of Gastroenterology and Hepatology, Oregon Health and Science University, Portland, Oregon, USA.

**Keywords:** CRC-AIM, cost-effectiveness analysis, colorectal cancer screening, simulation model, outreach, early detection of cancer

## Abstract

Outreach, including patient navigation, has been shown to increase the uptake of colorectal cancer (CRC) screening in underserved populations. This analysis evaluates the cost-effectiveness of triennial multi-target stool DNA (mt-sDNA) versus outreach, with or without a mailed annual fecal immunochemical test (FIT), in a Medicaid population. A microsimulation model estimated the incremental cost-effectiveness ratio using quality-adjusted life years (QALY), direct costs, and clinical outcomes in a cohort of Medicaid beneficiaries aged 50–64 years, over a lifetime time horizon. The base case model explored scenarios of either 100% adherence or real-world reported adherence (51.3% for mt-sDNA, 21.1% for outreach with FIT and 12.3% for outreach without FIT) with or without real-world adherence for follow-up colonoscopy (66.7% for all). Costs and outcomes were discounted at 3.0%. At 100% adherence to both screening tests and follow-up colonoscopy, mt-sDNA costed more and was less effective compared with outreach with or without FIT. When real-world adherence rates were considered for screening strategies (with 100% adherence for follow-up colonoscopy), mt-sDNA resulted in the greatest reduction in incidence and mortality from CRC (41.5% and 45.8%, respectively) compared with outreach with or without FIT; mt-sDNA also was cost-effective versus outreach with and without FIT ($32,150/QALY and $22,707/QALY, respectively). mt-sDNA remained cost-effective versus FIT, with or without outreach, under real-world adherence rates for follow-up colonoscopy. Outreach or navigation interventions, with associated real-world adherence rates to screening tests, should be considered when evaluating the cost-effectiveness of CRC screening strategies in underserved populations.

## Introduction

Colorectal cancer (CRC) is the second most common cause of cancer deaths; screening can reduce both the incidence of and death from CRC.^1^ US Preventive Services Taskforce (USPSTF) recommendations include both invasive (colonoscopy) and noninvasive (fecal immunochemical test [FIT], multi-target stool DNA [mt-sDNA]) screening alternatives. Although the prevalence of up-to-date screening with any recommended test among individuals aged 50 years and older has increased over time,^2^ reducing the risk of death from CRC requires repeat screening for individuals with normal test results and additional evaluation through follow-up colonoscopy of individuals with abnormal test results.^3^ Inequalities in uptake of screening exist and differences in access, use, and quality of screening may contribute to disparities in CRC death rates.^4^

Optimizing the quality of CRC screening depends on increasing the uptake of and follow-up to all aspects of the CRC screening continuum, from completion of initial tests to follow-up colonoscopy.^5^ Delays or failures in completing a follow-up colonoscopy have been associated with an increased risk of CRC diagnosis and mortality.^6^ Although provider-related (a lack of recommendation for screening) and patient-related barriers (fear, embarrassment, bowel preparation, cost, and lack of transportation) may impede screening,^4^ these barriers may be exacerbated in underserved populations, such as Medicaid enrollees.^7^ Outreach interventions, including patient navigation or reminders, may address these barriers by either facilitating the screening process or engaging patients and providers to increase adherence.^8^

All guideline-recommended CRC screening alternatives have been demonstrated to be cost-effective compared with no screening.^9^ However, noninvasive screening alternatives, such as FIT and mt-sDNA, can be done at home and do not require the restriction of either diet or medication, nor cathartic preparation^10^; these options may increase the accessibility and uptake of screening. Furthermore, mt-sDNA incorporates patient navigation to facilitate completion within its test. Although FIT does not inherently include outreach, programs and practices may choose to incorporate their own outreach strategy when providing FIT as an option to increase adherence. This analysis evaluates the cost-effectiveness of noninvasive CRC screening modalities of mt-sDNA versus outreach, with and without FIT, in a simulated Medicaid population.

## Methods

### Model overview

The Colorectal Cancer and Adenoma Incidence and Mortality Microsimulation Model (CRC-AIM) models the natural sequence of adenoma detection to carcinoma progression in unscreened patients, and includes test-related attributes, such as patient adherence to the initial screening strategy and follow-up colonoscopy.^11^ Details of this CRC-AIM model have been published previously^12^ and demonstrate substantial cross-model validity to the other Cancer Intervention and Surveillance Modeling Network model (CISNET).^11^

In brief, adenomas may grow and transition to preclinical cancer, which may transition to symptomatic CRC. CRC screening facilitates the identification and removal of adenomas and potential early detection of preclinical CRC. The ability of a stool-based CRC screening test to detect an adenoma or preclinical CRC is dependent on test performance (ie, sensitivity) and test completion (ie, adherence). All patients with a positive noninvasive screening test undergo a follow-up colonoscopy. Patients with a history of adenomas of any size are assumed to undergo surveillance with colonoscopy; time to next surveillance colonoscopy is based on the latest recommendations for follow-up colonoscopy.^13^ Complications included in the model are serious gastrointestinal events, other gastrointestinal events, and cardiovascular events, in line with CISNET methodology.^14^

This model focuses on estimating the cost-effectiveness of triennial mt-sDNA and annual FIT, both of which are guideline-endorsed stool-based CRC screening strategies. Although an annual guaiac-based fecal occult blood test is also a guideline-endorsed stool-based screening strategy, it was not included in this analysis because of its lower test performance parameters compared with both FIT and mt-sDNA. All mt-sDNA orders include centralized patient navigation support (available 24 hours per day, 365 days per year in >240 languages by the Exact Sciences Laboratories customer support center).^15^ For annual FIT screening, outreach strategies vary across practices.

Therefore, two previously reported outreach scenarios were modeled: a mailed letter encouraging CRC screening completion along with instructions about how to obtain a FIT test (with no actual FIT test provided), and outreach through a mailed letter encouraging CRC screening completion along with a FIT test.^16^ The estimates of sensitivity and specificity for mt-sDNA and FIT screening are identical to those applied in previous CISNET modeling analyses, which were used to inform the 2016 USPSTF guideline recommendations and are presented by adenoma size and cancer stage in Supplementary Table S1.^14^

### Model population

This analysis is from the perspective of the Medicaid payer and assumes a cohort of 1 million patients aged 50–64 years, alive and free of clinically diagnosed CRC. Upon entry into Medicaid, all patients are considered eligible for regular screening. The model uses a lifetime time horizon; all costs and outcomes were discounted at 3.0%.^17^

### Cost inputs

This model only considers direct medical costs (Supplementary Table S2). The cost of mt-sDNA was obtained from the Centers for Medicare & Medicaid Services Clinical Laboratory Fee Schedule.^18^ Screening costs for outreach with FIT and FIT itself were obtained from a study that evaluated the cost-effectiveness of a mailed outreach letter with a FIT test in a Medicaid population.^19^ The cost of FIT from this source is consistent with the cost of FIT from the Centers for Medicare & Medicaid Services Clinical Laboratory Fee Schedule.

The screening cost of an outreach alone was assumed conservatively to be equivalent to the cost of a FIT test,^19^ given that program costs vary across jurisdictions and by type of outreach. The screening cost associated with colonoscopy was taken from a claims database study^20^; the cost of complications were sourced from a budget impact and cost-consequence study.^21^ CRC-related direct medical costs were stratified by stage and time since diagnosis.^22^ Costs are reported in 2021 US dollars and were inflated using the Medicaid-to-Medicare Fee Index.^23^

### Utility inputs

Utility inputs at baseline, adjusted for age, were based on EuroQoL-5D US population norms (Supplementary Table S3).^24^ Utility decrements for all colonoscopies and complications for colonoscopy also were incorporated.^25^ Utility loss was included for patients who get CRC and was stratified by both level of care stage of CRC stage (Stage I–III and Stage IV); these were applied on a per-patient per-year basis.^25^

### Modeled adherence rates

Adherence to screening strategies and follow-up colonoscopy impacts the cost-effectiveness of screening strategies^12^; therefore, 3 different sets of adherence rates were considered in these analyses. In Analysis 1, it was assumed that patients were 100% adherent to both stool-based screening and follow-up colonoscopy.

Analysis 2 uses real-world screening estimates for stool-based screening strategies and assumes 100% adherence to follow-up colonoscopy (Table 1).

**Table 1.** Adherence Parameters Used in the Model for Base Case Analyses

Analysis	Screening test (%)	Reference	Follow-up colonoscopy (%)	Reference
Analysis 1				
mt-sDNA	100	Assumption	100	Assumption
Outreach with FIT	100	Assumption	100	Assumption
Outreach without FIT	100	Assumption	100	Assumption
Analysis 2				
mt-sDNA	51.3	Miller-Wilson et al^26^	100	Assumption
Outreach with FIT	21.1	Brenner et al^16^	100	Assumption
Outreach without FIT	12.3	Brenner et al^16^	100	Assumption
Analysis 3				
mt-sDNA	51.3	Miller-Wilson et al^26^	66.7	Assumed same as FIT
Outreach with FIT	21.1	Brenner et al^16^	66.7	Brenner et al^16^
Outreach without FIT	12.3	Brenner et al^16^	66.7	Brenner et al^16^

FIT, fecal immunochemical test; mt-sDNA, multi-target stool DNA.

Reported real-world adherence rates for mt-sDNA were taken from a cross-sectional study where adherence to mt-sDNA was assessed among Medicaid beneficiaries (*n* = 26,132) who, once determined eligible for screening by mt-sDNA, had a test ordered directly by their provider that was then shipped to the order-specified address by Exact Sciences Laboratories.^26^ Reported real-world adherence rates for outreach with or without FIT were taken from a randomized controlled trial assessing adherence among Medicaid beneficiaries (*n* = 2144).^16^

Analysis 3 uses real-world screening rates for stool-based screening plus real-world screening rates for follow-up colonoscopy. As real-world adherence data for follow-up colonoscopy in the Medicaid population were not available for mt-sDNA, follow-up colonoscopy rates from the randomized controlled trial assessing the outreach letter with and without FIT were used for all.^16^

Adherence to all surveillance and symptom colonoscopies was assumed to be 100% in all scenarios modeled. All adherence parameters were varied ±20% in sensitivity analyses.

### Cost-effectiveness analysis

The incremental cost-effectiveness ratio (ICER) of mt-sDNA versus outreach with or without FIT was the primary outcome. Secondary outcomes include clinical outcomes (either benefits or harms of CRC screening) and are presented as totals over the model lifetime per 1000 patients. Total lifetime CRC screening costs (including the cost of screening strategies, follow-up colonoscopies, complications, and program costs) are presented along with quality-adjusted life years (QALYs). There is no official willingness-to-pay (WTP) threshold in the United States; however, ICERs <$100,000 per QALY are considered to provide good value.^27^ As such, a WTP threshold of $100,000 per QALY was assumed.

### Scenario analyses

Across the 3 base case models (Analysis 1, 2, and 3), patients were assumed to have fixed cross-sectional adherence rates over time. Furthermore, patients who were nonadherent to follow-up colonoscopy were assumed to be nonadherent until they became symptomatic. Two alternate scenarios to these assumptions were explored.

#### Capped adherence over time

Data from the National Health Interview Survey (NHIS) suggests that up to 62.4% of individuals are up-to-date for CRC screening. A scenario analysis was explored using a calibration factor to reflect the fact that overall adherence to colonoscopy over 10 years may not reach 100%.^28^ Considering a base case of 38% adherence for colonoscopies in a given year,^29^ noncompliant individuals are offered screening every year at a constant declining rate (*r*) to ensure that these individuals remain noncompliant: 38% × (1 − *r*)^*t*^, if colonoscopy is delayed by *t* years, where 0 ≤ *t* ≤ 10. The result is a capped adherence rate where simulated individuals who are compliant with CRC screening match the reported NHIS rate (62.4%).^30^ The derived calibration factor is applied to all stool-based screening strategies to simulate a capped adherence scenario approach for Analysis 2 and 3.

#### Adherence extension for follow-up colonoscopy

In the base case models, patients who were nonadherent to follow-up colonoscopy were assumed to remain nonadherent until symptomatic. Scenario analyses were explored in which patients who did not undergo their follow-up colonoscopy initially were offered the follow-up colonoscopy in the following year, before being considered nonadherent. This scenario was explored for Analysis 3 under both the base case scenario and the capped adherence over time scenario.

### Sensitivity analyses

Sensitivity analyses were undertaken to account for uncertainty around model parameters and to test the robustness of the models. One-way sensitivity analyses for Analysis 3 in the base case were conducted varying adherence inputs 20% and costs and utility inputs ±10% from their base case value. A probabilistic sensitivity analysis also was conducted, in which all parameters are varied simultaneously at the same rates as the 1-way sensitivity analysis.

Threshold analyses were undertaken to evaluate the incremental cost-effectiveness of mt-sDNA versus outreach with or without FIT across various adherence rates. The threshold analyses presented the ICER for 2 different scenarios. In the first scenario, screening test adherence rates were varied from 0% to 100% and adherence to follow-up colonoscopy was held constant at 100%. In the second scenario, adherence to the screening test was held constant at 51.3% for mt-sDNA,^26^ 21.1% for outreach + FIT,^16^ and 12.3% for outreach alone,^16^ whereas adherence to follow-up colonoscopy was varied from 0% to 100%.

## Results

### Base case

When 100% adherence is assumed for both the screening test and follow-up colonoscopy (Analysis 1), outreach with or without FIT results in greater reductions in incidence (51.9% for both) and mortality (57.0% for both) compared with mt-sDNA (46.2% and 51.0%, respectively) (Table 2). When reported real-world adherence rates are considered for screening tests (Analysis 2), mt-sDNA results in a greater reduction in both the incidence and mortality from CRC, when compared with outreach with or without FIT (Table 2). When considering reported real-world adherence rates for both screening tests and follow-up colonoscopy (Analysis 3), mt-sDNA, compared with outreach with or without FIT still results in the greatest reduction in both the incidence and mortality from CRC; the magnitude of reduction in Analysis 3, however, is less than in Analysis 2 (Table 2).

**Table 2.** Discounted Clinical Outcomes Per 1000 Patients

Analysis	Screening strategy	No. of screening tests	Total number of COLs^a^	No. of CXs	No. of CRC cases	No. of CRC deaths	LYG	Incidence reduction (%)	Mortality reduction (%)
Analysis 1	None	0	80	1.7	79.8	36.0	0.00	0	0
	mt-sDNA	3904	1032	5.9	42.9	17.6	228.1	46.2	51.0
	Outreach + FIT	10,594	1107	6.5	38.4	15.5	251.1	51.9	57.0
	Outreach alone	10,594	1107	6.5	38.4	15.5	251.1	51.9	57.0
Analysis 2	None	0	80	2.3	79.8	36.0	0.00	0	0
	mt-sDNA	3205	894	5.9	46.7	19.5	203.5	41.5	45.8
	Outreach + FIT	2755	425	4.1	63.0	27.1	112.5	21.1	24.6
	Outreach alone	1660	290	3.4	69.2	30.3	73.2	13.3	15.7
Analysis 3	None	0	80	3.1	79.8	36.0	0.00	0	0
	mt-sDNA	3163	611	4.7	58.0	25.2	132.8	27.3	30.0
	Outreach + FIT	2772	301	3.4	68.8	30.0	76.3	13.9	16.5
	Outreach alone	1653	215	2.8	72.8	32.3	48.4	8.8	10.2

^a^ Total COLs include follow-up colonoscopies, surveillance colonoscopies, and colonoscopies for symptoms.

COL, colonoscopy; CRC, colorectal cancer; CX, complication; LYG, life years gained.

**FIG. 1.**
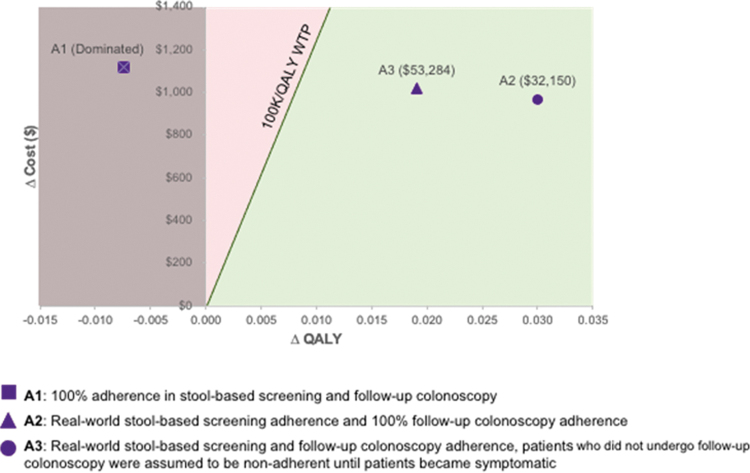
Incremental cost-effectiveness plane: mt-sDNA versus outreach + FIT. FIT, fecal immunochemical test; mt-sDNA, multi-target stool DNA; QALY, quality-adjusted life year.

Discounted total costs and QALYs are presented in Table 3. Lifetime costs were highest for mt-sDNA compared with outreach with or without FIT across all 3 analyses, ranging from $3837 in Analysis 2 to $4111 in Analysis 3, in part because of the screening test costs of mt-sDNA. However, mt-sDNA also resulted in the highest QALYs in Analysis 2 and 3 (Table 3). In the base case, compared with outreach + FIT, mt-sDNA is dominated (costs more and is less effective) in Analysis 1 but is cost-effective in Analysis 2 ($32,150/QALY) and Analysis 3 ($53,284/QALY) (Fig. 1). Similar results are observed for mt-sDNA versus outreach alone in the base case scenarios: mt-sDNA is dominated in Analysis 1 and is cost-effective versus outreach alone in Analysis 2 ($22,707/QALY) and Analysis 3 ($38,626/QALY) (Supplementary Fig. S1).

**Table 3.** Discounted Total Costs and Quality-Adjusted Life Years

Analysis	Screening strategy	Discounted costs					Discounted utilities			
		Screening	CXs	Program	CRC	Lifetime^a^	Screening disutility	CXs disutility	CRC disutility	QALY
Analysis 1	None	$42	$7	$0	$3041	$3091	−0.0002	−0.00002	−0.0346	15.100
	mt-sDNA	$2498	$34	$0^b^	$1580	$4111	−0.0041	−0.00012	−0.0214	15.174
	Outreach + FIT	$992	$38	$550	$1411	$2991	−0.0043	−0.00013	−0.0201	15.182
	Outreach alone	$992	$38	$0	$1411	$2440	−0.0043	−0.00013	−0.0201	15.182
Analysis 2	None	$42	$10	$0	$3041	$3093	−0.0002	−0.00003	−0.0346	15.100
	mt-sDNA	$2046	$34	$0^b^	$1757	$3837	−0.0034	−0.00011	−0.0233	15.166
	Outreach + FIT	$338	$21	$141	$2372	$2872	−0.0015	−0.00007	−0.0295	15.136
	Outreach alone	$221	$17	$0	$2625	$2862	−0.0010	−0.00006	−0.0316	15.123
Analysis 3	None	$42	$13	$0	$3041	$3097	−0.0002	−0.00005	−0.0346	15.100
	mt-sDNA	$1813	$25	$0^b^	$2192	$4030	−0.0023	−0.00009	−0.0271	15.143
	Outreach + FIT	$246	$17	$142	$2608	$3013	−0.0011	−0.00006	−0.0314	15.123
	Outreach alone	$166	$13	$0	$2771	$2951	−0.0007	−0.00005	−0.0327	15.115

^a^ Lifetime costs include colonoscopy costs, screening costs (stool-based screening costs and follow-up/symptom/surveillance colonoscopies, as relevant), cost of complications, cost of program, and cost of CRC.

^b^ Patient navigation program cost is included in the cost of screening for mt-sDNA.

QALY, quality-adjusted life-year.

### Scenario analyses

When screening rates over time are capped, the ICER for mt-sDNA versus outreach + FIT decreases to $15,561 (Analysis 2) and $27,306 (Analysis 3), representing greater cost-effectiveness. Similar results are observed for mt-sDNA versus outreach alone: the ICER decreases to $14,128 (Analysis 2) and $25,154 (Analysis 3). When patients are given an additional year to be considered adherent to follow-up colonoscopy, the ICER for mt-sDNA versus outreach + FIT using the inputs for Analysis 3 is $36,274 in the base case and $19,227 in the capped adherence scenario. Similar results are observed for mt-sDNA versus outreach alone: using the inputs for Analysis 3, giving patients an additional year to be considered adherent results in an ICER of $26,515 for the base case and $17,053 for the capped adherence scenario.

### Sensitivity analyses

One-way sensitivity analyses for mt-sDNA versus outreach (with or without FIT) found that adherence to follow-up colonoscopy across screening strategies had the largest impact on the ICER when real-world reported adherence rates were considered (Analysis 3) (Supplementary Figs. S2 and S3).

**FIG. 2.**
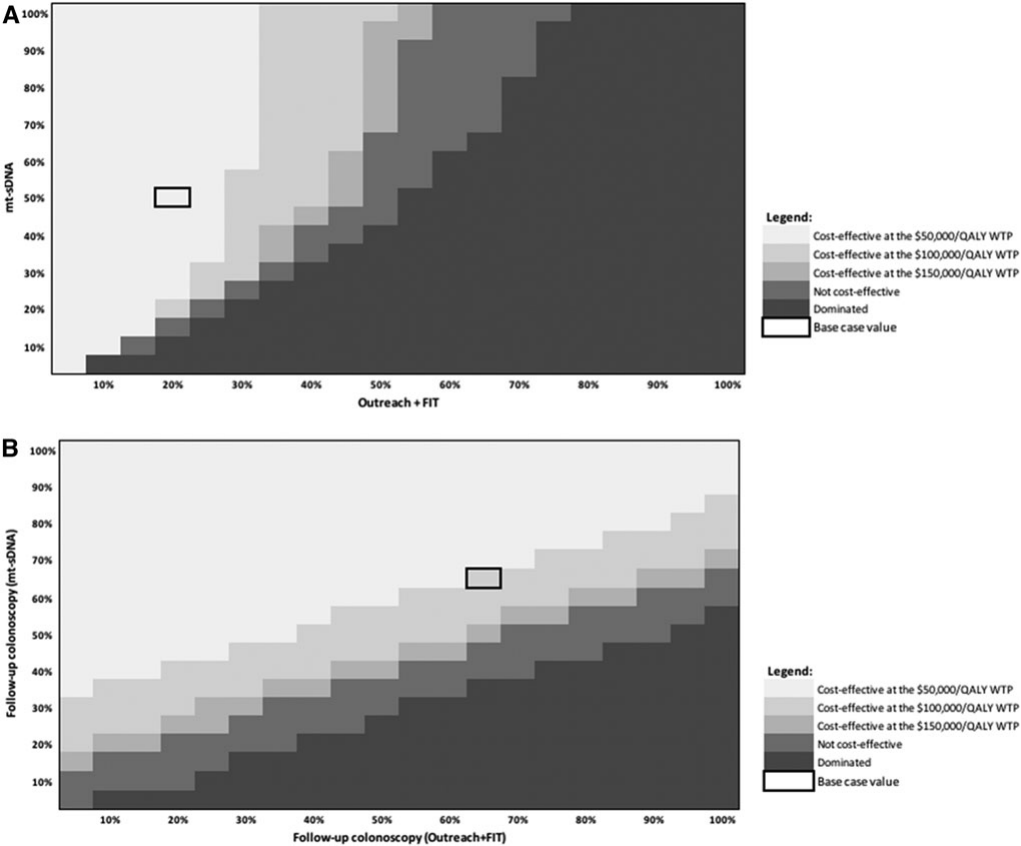
**(A)** Heatmap of mt-sDNA versus outreach + FIT when varying screening test adherence and follow-up colonoscopy adherence fixed.**(B)** Heatmap of mt-sDNA versus outreach + FIT when varying follow-up colonoscopy adherence (screening test rates fixed). 51.3% mt-sDNA adherence and 21.1% outreach + FIT adherence.

Figure 2A shows the cost-effectiveness of mt-sDNA versus outreach + FIT in the threshold analysis, where the adherence to stool-based screening is varied from 0% to 100% and follow-up colonoscopy adherence is fixed at 100%. At an adherence rate of 51.3% for mt-sDNA, adherence to outreach + FIT would need to exceed 40% to become cost-effective at a WTP of $100,000/QALY versus mt-sDNA (with follow-up colonoscopy fixed at 100%).

Figure 2B shows the cost-effectiveness of mt-sDNA versus outreach + FIT when adherence rates to the screening test are held constant (51.3% for mt-sDNA^26^ and 21.1% for outreach + FIT^16^), and the adherence to follow-up colonoscopy is varied from 0% and 100%. At an adherence rate of 66.7% to follow-up colonoscopy for mt-sDNA (equivalent to adherence to follow-up colonoscopy for FIT^16^), screening adherence to outreach + FIT would need to exceed 85% to be cost-effective versus mt-sDNA at a WTP of $100,000/QALY (with fixed screening adherence rates).

Similar results were found in the comparison of mt-sDNA with outreach alone (Supplementary Fig. S4A, B).

### Probabilistic analysis

In the probabilistic sensitivity analysis, mt-sDNA was cost-effective over outreach with or without FIT in 100% of the simulations at a WTP threshold of $100,000/QALY (Supplementary Fig. S3).

## Discussion

For CRC screening, a common perspective is “the best test is the test that gets done.”^8^ These results highlight the importance of outreach in increasing adherence and the associated impact on cost-effectiveness in an underserved population of Medicaid beneficiaries. At reported real-world rates of adherence, mt-sDNA is cost-effective compared with outreach through an outreach letter with or without FIT. Furthermore, mt-sDNA remains cost-effective when exploring alternate adherence rates across a wide range of assumptions. Programs can use these cost-effectiveness results to evaluate the impact of different screening tests and differing levels of adherence on CRC screening outcomes.

The study results are similar to others that have explored the cost-effectiveness of outreach strategies in CRC screening for underserved patients. A previous microsimulation model of Medicaid enrollees found that patient navigation had a greater impact on overall screening adherence rates compared to reminders alone or mailed FIT with a reminder.^31^ Unlike FIT, patient navigation through to completion of the screening test is integrated with every mt-sDNA test: patients are able to engage with the patient navigation system at all hours through telephone, kits are shipped directly to their home, and the kit includes a prepaid shipping label.^15^ Although adherence rates were higher when FIT was provided with an outreach letter than for an outreach letter alone, adherence to outreach + FIT would need to exceed 40% to be cost-effective versus mt-sDNA at adherence rates of 51.3%.

Multiple outreach strategies exist to increase adherence to CRC screening, including invitations from providers, reminder letters, telephone calls, and text messages, or reaching participants during other health care interactions.^32^ However, the absolute increase in screening associated with these interventions is variable. A randomized controlled trial that explored different forms of reminders for increasing adherence to FIT found that reminders included with a live call were more effective than written communication only; however, the estimated overall return rate of FIT remained <35% after the delivery of reminders.^33^

More encouragingly, a recent study found that when dual Medicaid/Medicare enrollees are first called to see if they would like to be mailed a FIT test, the completion rate among those who expressed interest was 68%.^34^ Further assessment of the reproducibility in an underserved population and scalability of this locally provided labor-intensive approach is needed before adoption as a broadly applicable strategy. Other interventions with FIT were found to be less effective in increasing screening rates. Among an underserved population composed largely of Medicaid beneficiaries, a randomized controlled trial of a text message only versus an opt-out option of receiving a FIT kit resulted in screening rates of 19.6%,^35^ whereas a separate randomized controlled trial comparing a text messaging and lottery incentive resulted in screening rates of 12.1%.^36^

A systematic review found that various interventions increased median FIT participation between 3.1% and 21.5%.^37^ The differences in the success of interventions to increase screening may be explained by multiple factors. O'Connor et al examined sociodemographic and health-related factors that moderate the effect of mailed FIT kit outreach in a cluster-randomized pragmatic trial in a primarily low-income population.^38^ While FIT completion was higher in the intervention group, compared with the control group, the only moderator with a statistically significant interaction was race, with persons of Asian descent having a 2-fold response to the intervention.^38^ The authors speculated that this may have been because of the wordless FIT instructions developed for the trial.^39^ This universal outreach may explain some of the success of mt-sDNA in a variety of populations: the patient navigation integrated within mt-sDNA is currently available in >240 languages.^15^

Data from this study analyses should be interpreted in the context of the assumptions. Although the screening test adherence inputs used in this model are based on real-world data, these data are based on a limited number of studies. However, the threshold analyses conducted allow cost-effectiveness end points to be correlated with a range of potential adherence rates. As no data were available for follow-up colonoscopy in patients who received mt-sDNA, a conservative assumption of equal adherence to follow-up colonoscopy was used; data in other populations indicate that follow-up to colonoscopy is higher among patients with a positive mt-sDNA than those with a positive FIT.^40^

Higher rates of FIT adherence have been observed in combination with outreach programs in non-Medicaid populations; these alternate adherence values and their impact on cost-effectiveness can be explored through this study's threshold analysis. Although a proxy cost of a FIT test was used for the outreach alone comparator, the cost used is conservative; an outreach strategy in clinical practice likely will cost more, which would increase the cost-effectiveness results herein. This model focused on noninvasive stool tests; however, barriers to completion of a test may be different for invasive tests such as colonoscopy.

This article did not explore factors associated with adherence; further study is needed to better understand the role of patient characteristics and preference on screening adherence behavior. Furthermore, although this article explores both cross-sectional and longitudinal adherence through the base case and scenario analyses, the impact of intermittent adherence on the cost-effectiveness of screening strategies should be analyzed in detail in future models.

## Conclusions

Being adherent to screening may reduce the risk of dying from CRC by >60% and increasing adherence to CRC screening strategies remains a key public health goal.^3^ Patient navigation or outreach interventions may increase adherence rates to screening tests. These interventions should be considered when evaluating the cost-effectiveness of CRC screening strategies to ensure that underserved populations have the best chance of achieving and maintaining adherence to screening, allowing the maximum clinical benefit of reduced incidence of and mortality from CRC.

## Supplementary Material

Supplemental data
