# Supplementary material for: Cost-Effectiveness of Outreach Strategies for Stool-Based Colorectal Cancer Screening in a Medicaid Population
Source: Popul Health Manag. 2022 Jun 7;25(3):343–51. doi: 10.1089/pop.2021.0185 (PMC9232231; doi:10.1089/pop.2021.0185)
Supplement: Supplemental data [file Suppl_Data.docx]

**Supplementary Table 1. Test performance parameters**

|  | Sensitivity | | | | | | | Specificity |
| --- | --- | --- | --- | --- | --- | --- | --- | --- |
|  | **Adenomas** | | | **Cancer** | | | |  |
|  | **<6mm** | **6-9mm** | **10mm+** | **Stage I** | **Stage II** | **Stage III** | **Stage IV** |  |
| mt-sDNA | 17.2% | 17.2% | 42.4% | 92.3% | 92.3% | 92.3% | 92.3% | 89.8% |
| FIT | 7.6% | 7.6% | 23.8% | 73.8% | 73.8% | 73.8% | 73.8% | 96.4% |

**Supplementary Table 2. Cost inputs^a^**

| Parameter | Base Value | Distribution range | Reference |
| --- | --- | --- | --- |
| Screening costs |  |  |  |
| Colonoscopy | $1,076.37 | ± 10% | Pyenson(1) |
| mt-sDNA | $508.87 | ± 10% | CMS Clinical Laboratory Fee Schedule |
| Outreach + FIT | $79.57 | ± 10% | Wheeler^a^ (2) |
| Outreach alone | $61.92 | ± 10% | Assumption |
| Complications |  |  |  |
| Gastrointestinal | $6,390.07 | ± 10% | Hathway(3) |
| Serious gastrointestinal | $18,217.03 | ± 10% |  |
| Cardiovascular | $8,192.46 | ± 10% |  |
| CRC costs |  |  |  |
| Stage I Initial Care | $26,995.85 | ± 10% | Naber(4) |
| Stage I Continuous Care | $2,174.02 | ± 10% |  |
| Stage I Terminal Care, CRC death | $55,333.35 | ± 10% |  |
| Stage I Terminal Care, non-CRC death | $13,522.62 | ± 10% |  |
| Stage II Initial Care | $38,284.53 | ± 10% |  |
| Stage II Continuous Care | $2,644.44 | ± 10% |  |
| Stage II Terminal Care, CRC death | $62,425.42 | ± 10% |  |
| Stage II Terminal Care, non-CRC death | $14,544.45 | ± 10% |  |
| Stage III Initial Care | $54,492.08 | ± 10% |  |
| Stage III Continuous Care | $4,257.35 | ± 10% |  |
| Stage III Terminal Care, CRC death | $64,588.91 | ± 10% |  |
| Stage III Terminal Care, non-CRC death | $20,234.13 | ± 10% |  |
| Stage IV Initial Care | $79,293.67 | ± 10% |  |
| Stage IV Continuous Care | $21,435.71 | ± 10% |  |
| Stage IV Terminal Care, CRC death | $80,365.70 | ± 10% |  |
| Stage IV Terminal Care, non-CRC death | $49,583.75 | ± 10% |  |

^a^Costs were inflated to 2021 US dollars (USD) using the Medicaid-to-Medicare Fee Index.(5)

^b^Cost includes materials for mailing, personnel costs, patient navigation and cost of FIT

**Supplementary Table 3. Utility inputs**

|  | Base value | | Distribution Range | Reference |
| --- | --- | --- | --- | --- |
| Health State Utility | EQ-5D population norms | | Not varied | Szende(6) |
| Per event |  | |  |  |
| Colonoscopy | -0.0055 | | ± 10% | Goede(7) |
| Complication from colonoscopy, any | -0.0384 | | ± 10% | Goede(7) |
| Per person-year of colorectal care | Stage I-III | Stage IV |  |  |
| Initial care | -0.15 | -0.34 | ± 10% | Goede(7) |
| Continuous care | -0.10 | -0.29 | ± 10% | Goede(7) |
| Terminal care, CRC death | -0.29 | -0.29 | ± 10% | Goede(7) |
| Terminal care, non-CRC death | -0.10 | -0.29 | ± 10% | Goede(7) |

**Supplemental Figure 1 – Incremental cost-effectiveness plane: mt-sDNA versus outreach alone**

*
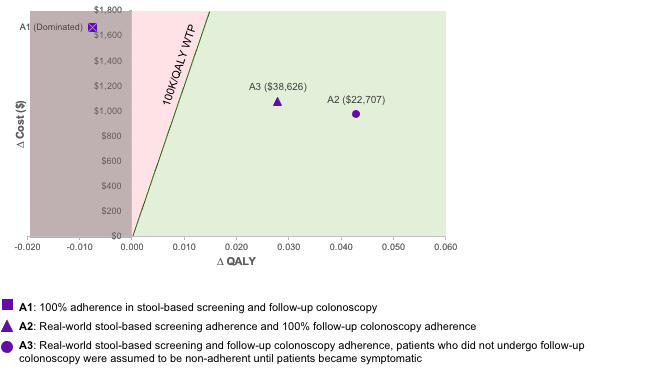
*

WTP: willingness-to-pay

**Supplemental Figure 2 – One way sensitivity analysis mt-sDNA versus outreach + FIT**


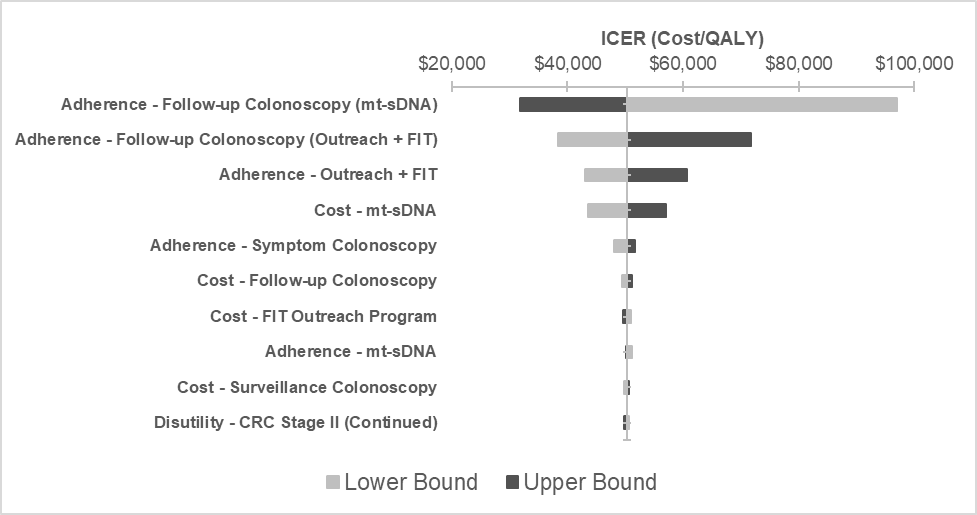


**Supplemental Figure 3 – One way sensitivity analysis mt-sDNA versus outreach alone**


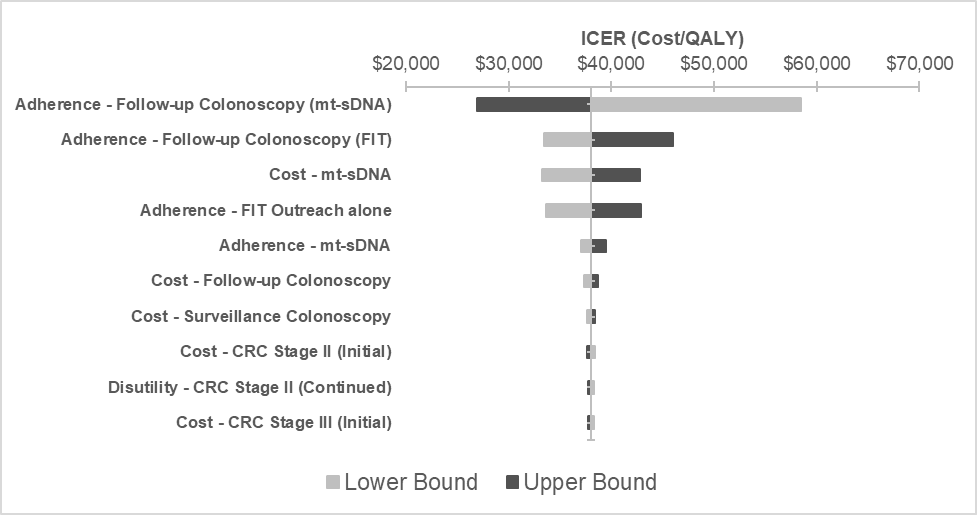


**Supplemental Figure 4A. Heatmap of mt-sDNA versus outreach alone when varying screening test adherence (follow-up colonoscopy adherence fixed)**

**
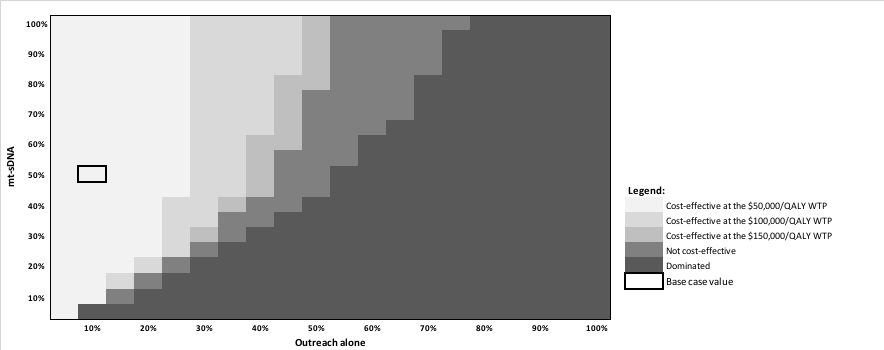
**

**Supplemental Figure 4B. Heatmap of mt-sDNA versus outreach alone when varying follow-up colonoscopy adherence (screening test rates fixed^a^)**


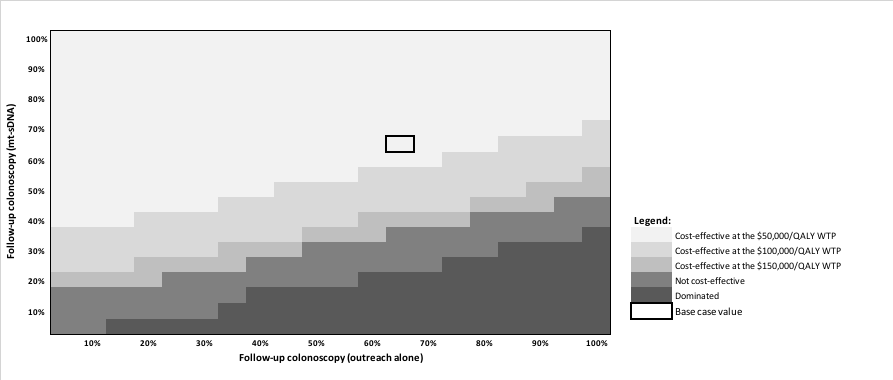


^a^51.3% mt-sDNA adherence and 12.3% FIT adherence

**Supplemental Figure 3. Probabilistic sensitivity analysis of mt-sDNA versus outreach with or without FIT after 500 iterations**

**
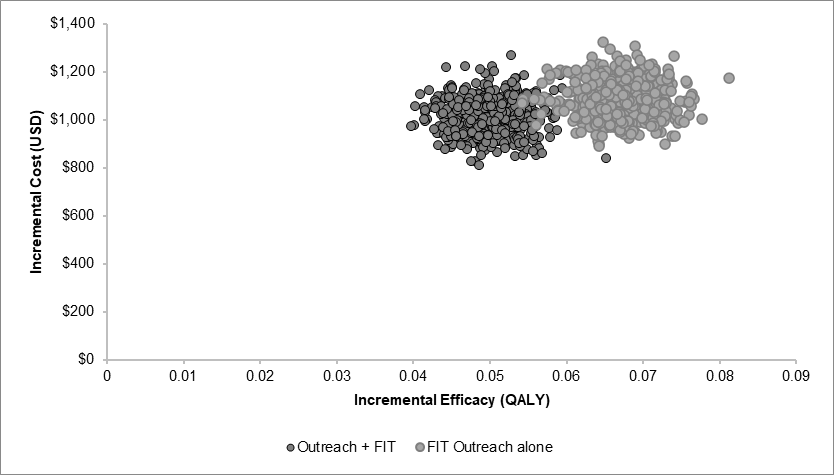
**

QALY: quality-adjusted life year; USD: United States dollars

1. Pyenson B, Scammell C, Broulette J. Costs and repeat rates associated with colonoscopy observed in medical claims for commercial and Medicare populations. BMC Health Serv Res. 2014;14:92.

2. Wheeler SB, O'Leary MC, Rhode J, Yang JY, Drechsel R, Plescia M, et al. Comparative cost-effectiveness of mailed fecal immunochemical testing (FIT)-based interventions for increasing colorectal cancer screening in the Medicaid population. Cancer. 2020;126(18):4197-208.

3. Hathway JM, Miller-Wilson LA, Jensen IS, Ozbay B, Regan C, Jena AB, et al. Projecting total costs and health consequences of increasing mt-sDNA utilization for colorectal cancer screening from the payer and integrated delivery network perspectives. J Med Econ. 2020;23(6):581-92.

4. Naber SK, Knudsen AB, Zauber AG, Rutter CM, Fischer SE, Pabiniak CJ, et al. Cost-effectiveness of a multitarget stool DNA test for colorectal cancer screening of Medicare beneficiaries. PLoS One. 2019;14(9):e0220234.

5. United States Department of Labor. CPI for All Urban Consumers, Medical care services [Available from: <https://beta.bls.gov/dataViewer/view/timeseries/CUSR0000SAM2>.

6. In: Szende A, Janssen B, Cabases J, editors. Self-Reported Population Health: An International Perspective based on EQ-5D. Dordrecht (NL): Springer; 2014.

7. Goede SL, Rabeneck L, van Ballegooijen M, Zauber AG, Paszat LF, Hoch JS, et al. Harms, benefits and costs of fecal immunochemical testing versus guaiac fecal occult blood testing for colorectal cancer screening. PLoS One. 2017;12(3):e0172864.
